# Enhanced B-Cell Receptor Recognition of the Autoantigen Transglutaminase 2 by Efficient Catalytic Self-Multimerization

**DOI:** 10.1371/journal.pone.0134922

**Published:** 2015-08-05

**Authors:** Jorunn Stamnaes, Rasmus Iversen, M. Fleur du Pré, Xi Chen, Ludvig M. Sollid

**Affiliations:** Centre for Immune Regulation and Department of Immunology, University of Oslo and Oslo University Hospital, Oslo, Norway; Tulane University, UNITED STATES

## Abstract

A hallmark of the gluten-driven enteropathy celiac disease is autoantibody production towards the enzyme transglutaminase 2 (TG2) that catalyzes the formation of covalent protein-protein cross-links. Activation of TG2-specific B cells likely involves gluten-specific CD4 T cells as production of the antibodies is dependent on disease-associated HLA-DQ allotypes and dietary intake of gluten. IgA plasma cells producing TG2 antibodies with few mutations are abundant in the celiac gut lesion. These plasma cells and serum antibodies to TG2 drop rapidly after initiation of a gluten-free diet, suggestive of extrafollicular responses or germinal center reactions of short duration. High antigen avidity is known to promote such responses, and is also important for breakage of self-tolerance. We here inquired whether TG2 avidity could be a feature relevant to celiac disease. Using recombinant enzyme we show by dynamic light scattering and gel electrophoresis that TG2 efficiently utilizes itself as a substrate due to conformation-dependent homotypic association, which involves the C-terminal domains of the enzyme. This leads to the formation of covalently linked TG2 multimers. The presence of exogenous substrate such as gluten peptide does not inhibit TG2 self-cross-linking, but rather results in formation of TG2-TG2-gluten complexes. The celiac disease autoantibody epitopes, clustered in the N-terminal part of TG2, are conserved in the TG2-multimers as determined by mass spectrometry and immunoprecipitation analysis. TG2 multimers are superior to TG2 monomer in activating A20 B cells transduced with TG2-specific B-cell receptor, and uptake of TG2-TG2-gluten multimers leads to efficient activation of gluten-specific T cells. Efficient catalytic self-multimerization of TG2 and generation of multivalent TG2 antigen decorated with gluten peptides suggest a mechanism by which self-reactive B cells are activated to give abundant numbers of plasma cells in celiac disease. Importantly, high avidity of the antigen could explain why TG2-specific plasma cells show signs of an extrafollicular generation pathway.

## Introduction

Celiac disease is a prevalent enteropathy with autoimmune features including highly disease-specific autoantibodies to the enzyme transglutaminase 2 (TG2) and selective immune killing of enterocytes [1]. The disease is driven by a response to cereal gluten proteins, and the small intestinal lesion and the autoantibodies disappear when gluten is eliminated from the diet. The lesion is characterized by villus blunting, plasma cell infiltration and also by presence of gluten-specific CD4 T cells which respond to gluten epitopes presented by the disease-associated MHC class II molecules HLA-DQ2.5, HLA-DQ2.2 and HLA-DQ8. These T cells recognize post-translationally modified gluten peptides with certain glutamine residues converted to glutamate. This modification is mediated by the same enzyme to which there are autoantibodies—TG2.

TG2 is a ubiquitously expressed enzyme which is allosterically regulated by Ca^2+^ and guanosine-5’-triphosphate (GTP) [2]. GTP-bound TG2 adopts a closed, inactive conformation whereas Ca^2+^-bound TG2 adopts an open, extended conformation that is catalytically active. TG2 selectively modifies glutamine residues by hydrolysis to form glutamate (deamidation) or by cross-linking the glutamine side chain either to the side chain amino group of lysine residues or to small, biogenic primary amines (transamidation) [2]. Peptide glutamine targeting by TG2 is sequence-dependent with preference for glutamine residues in the sequence QXP [3, 4]. This motif is often found in gluten peptides, and many gluten peptides are excellent substrates for TG2. Among the many thousand peptides present in a digest of gluten, the preferred substrates for TG2 are the peptides that are recognized by celiac disease T cells suggesting that the enzyme is involved in the selection of pathogenic T-cell epitopes [5].

IgA antibodies towards TG2 and deamidated gluten serve as serological markers for diagnosis of celiac disease [6–8]. These tests are only useful in subjects who eat gluten, as the antibodies disappear from the circulation within few months after commencement of a gluten-free diet [9, 10]. Anti-TG2 autoantibodies are only observed in individuals who carry HLA-DQ2.5, HLA-DQ2.2 or HLA-DQ8 [11]. Activation of auto-reactive B cells thus appears to involve gluten and the celiac disease-associated MHC class II molecules. Conceivably, gluten-specific T cells may be involved in the breaking of self-tolerance to TG2 by providing help to TG2-specific B cells [12]. In support of this model, it has been demonstrated that TG2 can covalently cross-link gluten peptides harboring T-cell epitopes to itself creating TG2-gluten complexes [13].

We have recently characterized the anti-TG2 antibody response of celiac disease lesions by staining of antigen-specific plasma cells. In the active lesion, on average 10% of the plasma cells are TG2-specific [14], but after commencement of a gluten-free diet these specific plasma cells rapidly drop in numbers [15]. Sequencing of immunoglobulin genes and generation of recombinant monoclonal antibodies of single TG2-specific IgA+ plasma cells revealed that the antibodies have biased and limited VH gene-segment usage and few somatic mutations [14]. The same characteristics were also observed for antibodies cloned from IgA+ plasma cells specific for deamidated gluten. The low degree of somatic mutations suggests that the B-cell responses to deamidated gluten and TG2 have shared mechanistic origins [16]. The VH gene-segment usage of anti-TG2 antibodies reflects their targeting of epitopes of TG2. Four major epitopes were identified, and they all cluster in the N-terminal part of TG2 [17]. Reversion of the antibody mutations to presumed germ line configuration led to a decrease in affinity in a panel of anti-TG2 antibodies, suggesting that the antibodies had undergone affinity maturation but to a limited degree. Together with the short-lived duration of the anti-TG2 response, this could indicate that the anti-TG2 antibody response develops extrafollicularly.

If the antibody response to TG2 in celiac disease develops extrafollicularly, what dictates this? One possibility is that TG2-mediated cross-linking of IgD B-cell receptor (BCR) molecules on non-germinal center B cells is involved in the activation of TG2-specific B cells [14] (Iversen R et al, *manuscript submitted*). Alternatively, extrafollicular B-cell activation could be a consequence of the physical appearance of the antigen, as high antigen avidity is known to promote extrafollicular B-cell responses and increase plasma cell generation (reviewed in [18]). We thus asked if there might be situations where TG2 avidity is increased. We here report that TG2 through conformation-dependent homotypic association acts as a self-substrate and forms multivalent covalently linked self-complexes even in the presence of excess competitor substrate. Such multivalent TG2 complexes can be recognized by celiac anti-TG2 monoclonal antibodies, and we demonstrate that TG2 multimers compared to monomer efficiently activate A20 B cells transduced with TG2-specific BCR. It is known that multivalent antigens are particularly effective at activating B cells [19, 20] and, of relevance to celiac disease, at breaking B-cell tolerance especially when coupled with appropriate T-cell help [21]. We demonstrate that peptides harboring gluten T-cell epitopes could be cross-linked to TG2 multimers, and these were effective antigens for B cell-mediated activation of gluten-specific T cells. Our findings describe unique properties of TG2 that shed new light on how this self-protein may trigger the massive expansion of self-reactive plasma cells with few mutations observed in celiac disease.

## Results

### TG2 serves as a good substrate for itself even when substrate competitors are in excess

**Fig 1:**
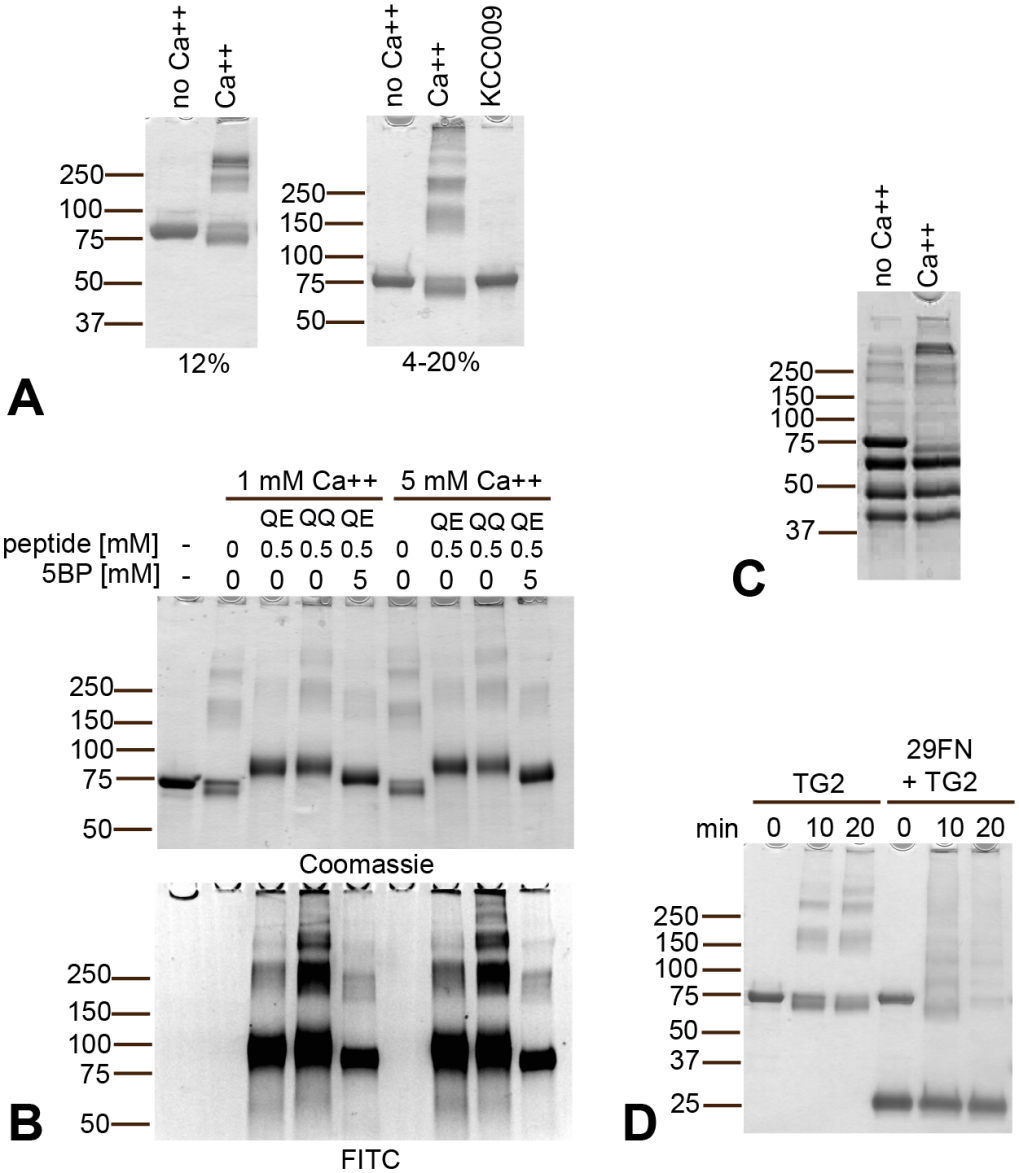
TG2 self-crosslinking in the absence or presence of competitor substrate. (A) TG2 (2 μM) incubated in the presence of CaCl_2_ will cross-link itself into complexes that can be resolved by gradient SDS-PAGE (4–20%) but not by standard 12% SDS-PAGE. Self-crosslinking requires TG2 catalytic activity and does not happen in the absence of CaCl_2_ or presence of an active site inhibitor. (B) TG2 (1 μM) self-crosslinking as seen by gradient SDS-PAGE (at 15min) occurs even in the presence of a high excess of glutamine donor substrate (DQ2.5-glia-α2-QQ-FITC QQ; DQ2.5-glia-α2-QE-FITC, QE) or primary amine (5BP) (Coomassie). Incorporation of the FITC-labeled peptides is observed both in TG2 monomer and TG2 multimers (FITC). (C) Incubation of TG2 (78 kDa) together with human albumin (64 kDa), human IgG (50 kDa + 25 kDa) and ovalbumin (45 kDa) in the presence of CaCl_2_ results in selective disappearance of the monomeric TG2 band, indicating that self-crosslinking is a specific event. (D) Incubation of TG2 with excess of the known protein substrate 29kDa FN fragment (29FN) results in disappearance of TG2 monomer and incorporation of both TG2 and 29FN into complexes.

Human recombinant TG2 incubated with Ca^2+^ serves as a cross-linking substrate to itself (Fig 1A). Self-crosslinked high molecular weight TG2 complexes could be resolved by gradient SDS-PAGE as distinct bands corresponding to monomer, dimer, trimer and higher-order multimers (Fig 1A). Importantly, we found that TG2 self-crosslinking also occurred in the presence of competing exogenous substrate as observed following incubation of TG2 with a high excess of gluten peptide substrate (DQ2.5-glia-α2-QE-FITC and DQ2.5-glia-α2-QQ-FITC with one or two targeted glutamine residues respectively) and the small primary amine 5-(biotinamido)pentylamine (5BP) (Fig 1B). Of note, gluten peptides were readily cross-linked to TG2 as visualized by FITC fluorescence, in line with previous observations [13]. However, not even a 500 fold excess of gluten peptide substrate and 5000 fold excess of 5BP prevented the parallel reaction of TG2 self-crosslinking. Rather, gluten peptides were concomitantly incorporated into TG2 multimers. The gluten peptide harboring two targeted glutamine residues was cross-linked to a larger extent to TG2 as seen by FITC fluorescence. A comparable degree of self-crosslinking was observed at *in vitro* saturating and physiological CaCl_2_ concentrations (5 mM and 1 mM respectively, Fig 1B). To determine the ability of TG2 to self-crosslink in the presence of other proteins, TG2 was incubated with a mix of proteins that could readily be distinguished by SDS-PAGE. In the presence of CaCl_2_, only the monomeric band of TG2 disappeared (Fig 1C). Thus, TG2 itself is the only protein in this mixture that serves as a cross-linking substrate. Incubation of TG2 with a known protein substrate, the 29kDa proteolytic fragment of fibronectin (FN) [22, 23] also led to a clear decrease in the amount of monomeric TG2 (Fig 1D). Here, several cross-linked products were generated likely consisting of a mix of TG2 multimers and TG2 cross-linked with one or more 29kDa FN fragments. In summary, TG2 self-crosslinking is a specific event that is not readily prevented even by large excess of competing substrates, indicating that TG2 serves as a preferred substrate for itself.

### Conformation-dependent homotypic association of TG2 explains susceptibility for self-crosslinking

**Fig 2:**
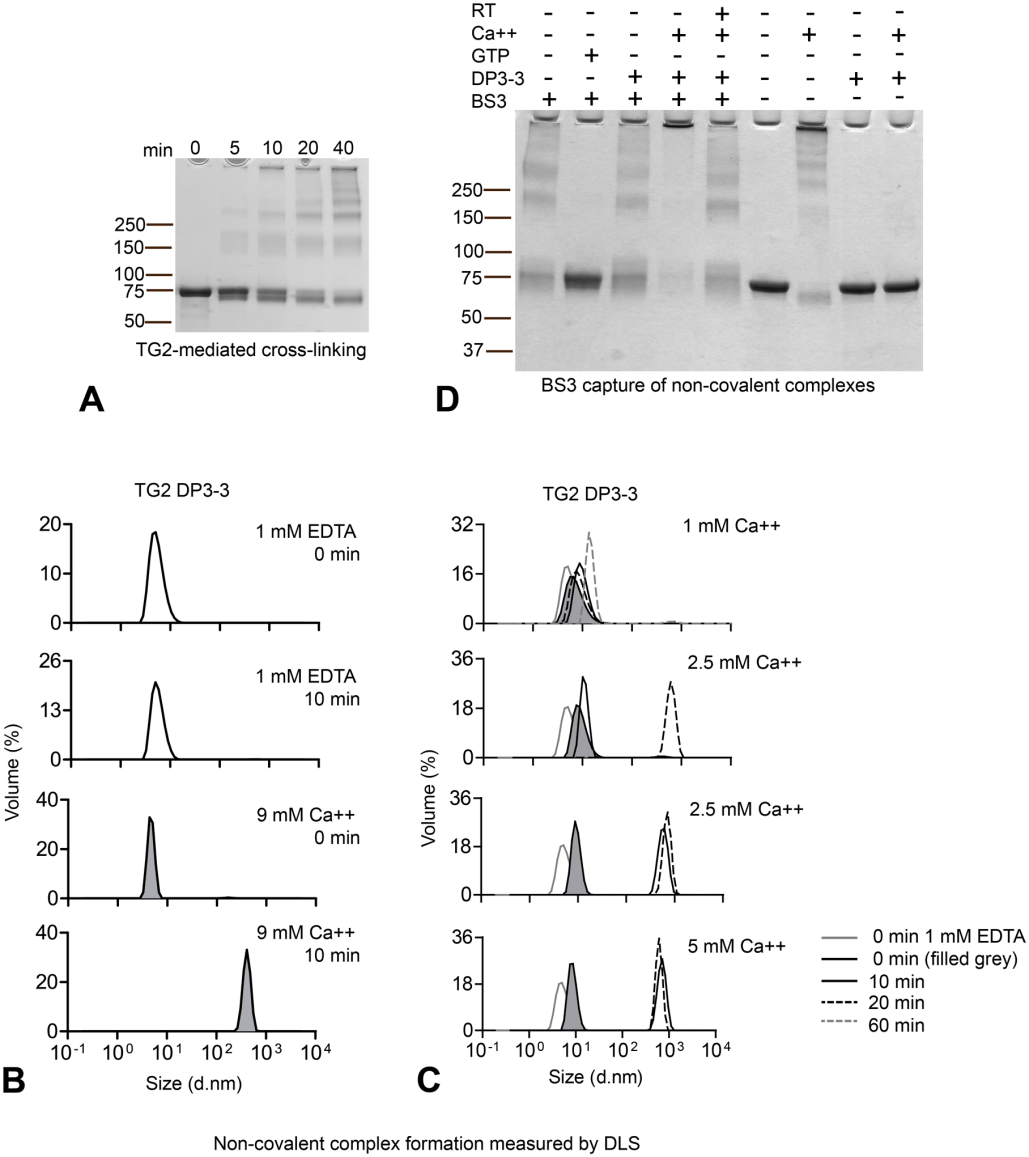
Homotypic TG2 association is conformation dependent (A) TG2 (2.4 μM) self-crosslinking over time occurs via initial formation of dimer and trimer as observed after 5 and 10 min. (B) Inactive, inhibitor bound TG2 produced in *E*. *coli* (TG2-DP3-3) was incubated at 37°C in the presence of 1 mM EDTA or 9 mM CaCl_2_ and aggregation was measured by DLS. The graphs display volume percentage (Y axis) and size as diameter (d. nm) on the X-axis. Incubation with 9 mM CaCl_2_ at 37°C results in aggregation formation already after 10 min. (C) Titration of CaCl_2_ was performed at 37°C and non-covalent complex formation was compared at different time-points: 0 min (black line, grey fill), 10 min (black solid line) and 20 min (black dotted line). For 1 mM CaCl_2_, 60 min incubation is also shown (grey dotted line). The solid grey line shows TG2-DP3-3 incubated in 1 mM EDTA at time 0 min for comparison. Two datasets from the same experiment are shown for 2.5 mM CaCl_2_ as variation was observed in the degree of high molecular weight multimer formation for this CaCl_2_ concentration. (D) Capture of non-covalent TG2-TG2 associations using BS3 cross-linking reagent. TG2 was incubated in the absence of effectors or together with 5 mM CaCl_2_ or 1 mM GTP for 30 min at 37°C or, where indicated, for 30 min at room temperature (RT) before addition of BS3. To avoid enzymatic self-crosslinking in the presence of Ca^2+^ the enzyme was pretreated with the active-site inhibitor DP3-3. Incubation of TG2-DP3-3 with CaCl_2_ at 37°C followed by BS3 treatment results in the formation of large complexes that do not migrate through the gel.

TG2 initially formed cross-linked dimers, extending into multimers over time (Fig 2A) suggesting that the complexes form in an orderly manner through specific TG2-TG2 interactions. To study non-covalent interactions we utilized a catalytically inactive variant of TG2 where the active site is covalently linked to the inhibitor Ac-P-DON-LPF-NH_2_ (DP3-3) (TG2-DP3-3). By dynamic light scattering (DLS) analysis we found that inactive TG2 incubated with EDTA at 37°C stayed as a monomer (Fig 2B). However, incubation with 9 mM CaCl_2_ at 37°C resulted in rapid formation of high molecular weight multimers (Fig 2B). Upon titration of CaCl_2_, we observed rapid multimer-formation also at 5 mM CaCl_2_ and at a slower rate at 2.5 mM CaCl_2_. Incubation with 1 mM CaCl_2_ resulted in a smaller increase in size, likely reflecting formation of low order complexes (Fig 2C). The resolving power of our DLS instrument did not allow us to determine whether this increase in size reflected formation of dimers or trimers. We therefore utilized the bivalent amine reactive cross-linker bis(sulfosuccinimidyl)suberate (BS3) which will covalently cross-link and capture non-covalent protein-protein interactions. BS3 treatment of inactive TG2 (TG2-DP3-3) pre-incubated with 5 mM CaCl_2_ at 37°C confirmed the formation of non-covalent high molecular weight multimers (Fig 2D) that we had observed by DLS. In addition, TG2 was indeed captured as dimers, trimers and lower order multimers when incubated at 37°C in the absence of CaCl_2_, or at room temperature with 5 mM CaCl_2_. Importantly, no self-assembly was observed when TG2 was incubated with GTP to induce the compact, closed conformation. Thus, TG2 can form non-covalent TG2-TG2 complexes depending on the conformational state of the enzyme. Binding of Ca^2+^ promotes this association, and saturating concentration of CaCl_2_ and incubation at 37°C shifts the interaction from transient dimer/trimer formation to formation of high molecular weight complexes.

### The C-terminal domains of TG2 are required for homotypic association and subsequent self-crosslinking

**Fig 3:**
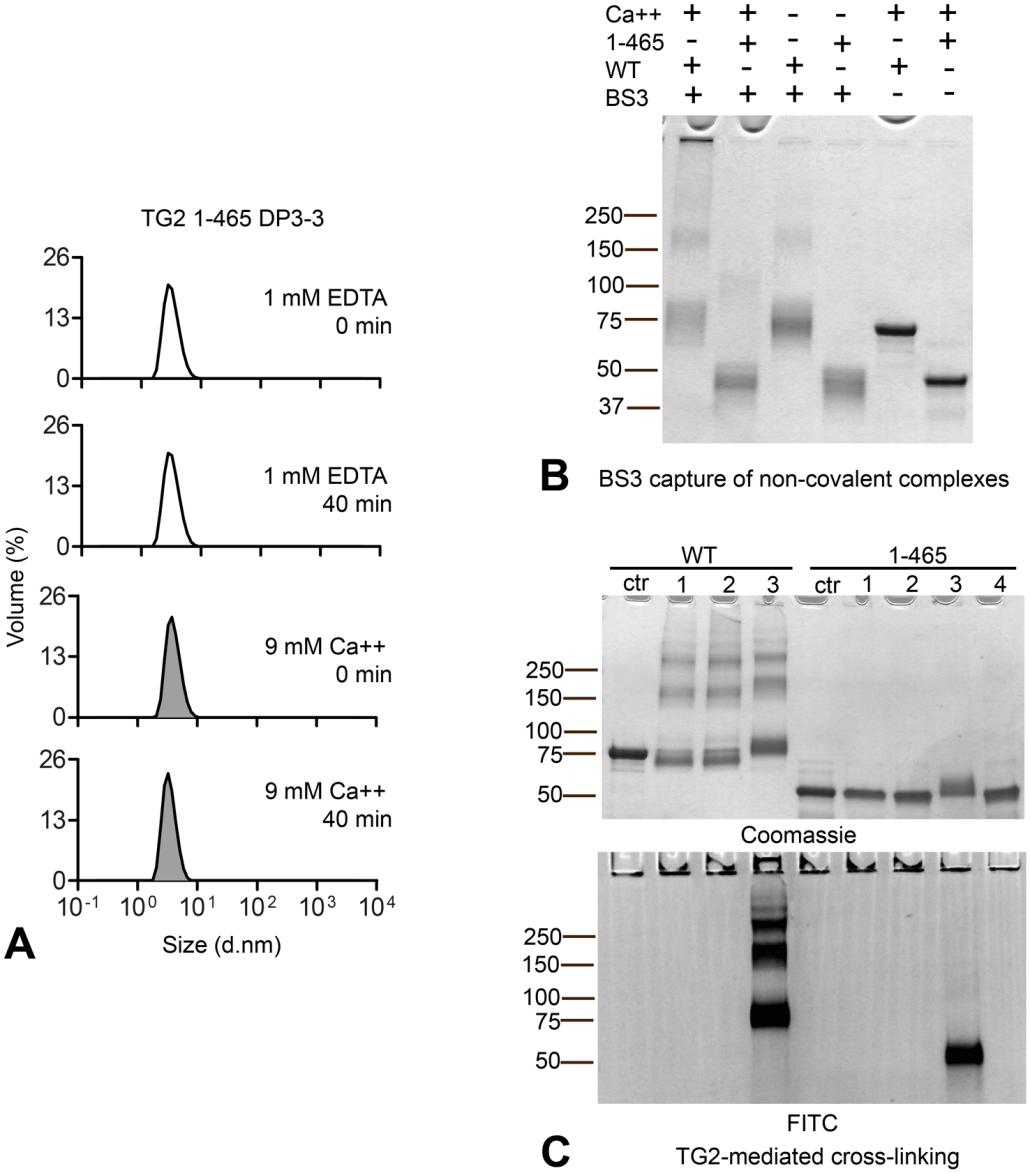
The C-terminal domains are required for homotypic association and self-crosslinking (A) A truncated variant of TG2 lacking the two C-terminal domains (1–465) was expressed in *E*. *coli* and treated with DP3-3. The inactive, inhibitor-bound deletion mutant (1-465-DP3-3) was incubated at 37°C in the presence of 1 mM EDTA or 9 mM CaCl_2_ and aggregation was measured by DLS. The graphs display volume percentage (Y axis) and diameter (d.nm) on the X-axis. No multimer formation of the truncated TG2 was observed following 40 min incubation. (B) Capture of non-covalent complexes of inactive *E*. *coli*-produced DP3-3-treated full length (WT) and 1–465 deletion mutant (1–465) TG2 following incubation at 37°C using BS3 as described in Fig 2C. No complex formation was observed for the deletion mutant compared to full-length TG2. (C) Incubation of active *E*. *coli*-produced deletion mutant (1–465) with CaCl_2_ did not result in self-crosslinking as observed for full length *E*. *coli*-produced TG2. The gel shows incubation of different enzyme concentrations with 5 mM CaCl_2_ at 37°C for 60 min: 1) 0.7 μM, 2) 1.4 μM, 4) 4.2 μM and 3) 1.4 μM in the presence of 0.2 mM DQ2.5-glia-α2-QQ-FITC. Incorporated gluten peptides are visualized by FITC fluorescence.

Comparing the crystal structures of closed, GTP bound TG2 and the open, presumably active conformation of TG2 [24, 25], the most notable structural change involves movement of the two C-terminal beta-barrel domains away from the catalytic core domain. To address if the C-terminal domains were directly involved in TG2 multimerization, we created a deletion mutant (1–465) lacking these two domains. Indeed, in contrast to full length TG2, an inactive inhibitor-bound form of this deletion mutant (1-465-DP3-3) did not form multimers upon incubation with 9 mM CaCl_2_ at 37°C as observed by DLS (Fig 3A) or by capture with BS3 (Fig 3B). Further, in its active state, this deletion mutant did not cross-link itself into covalent multimers as observed for full length TG2 despite being able to efficiently cross-link FITC-labeled gluten peptides to itself (Fig 3C). The two C-terminal domains of TG2 thus appear pivotal for homotypic TG2 association and also for self-crosslinking. This supports the notion that homotypic association is required for TG2 to efficiently act as a self-substrate.

### Glutamine and lysine residues involved in self-crosslinking are located in the C-terminal half of TG2 preserving epitopes of the N-terminal domain

**Fig 4:**
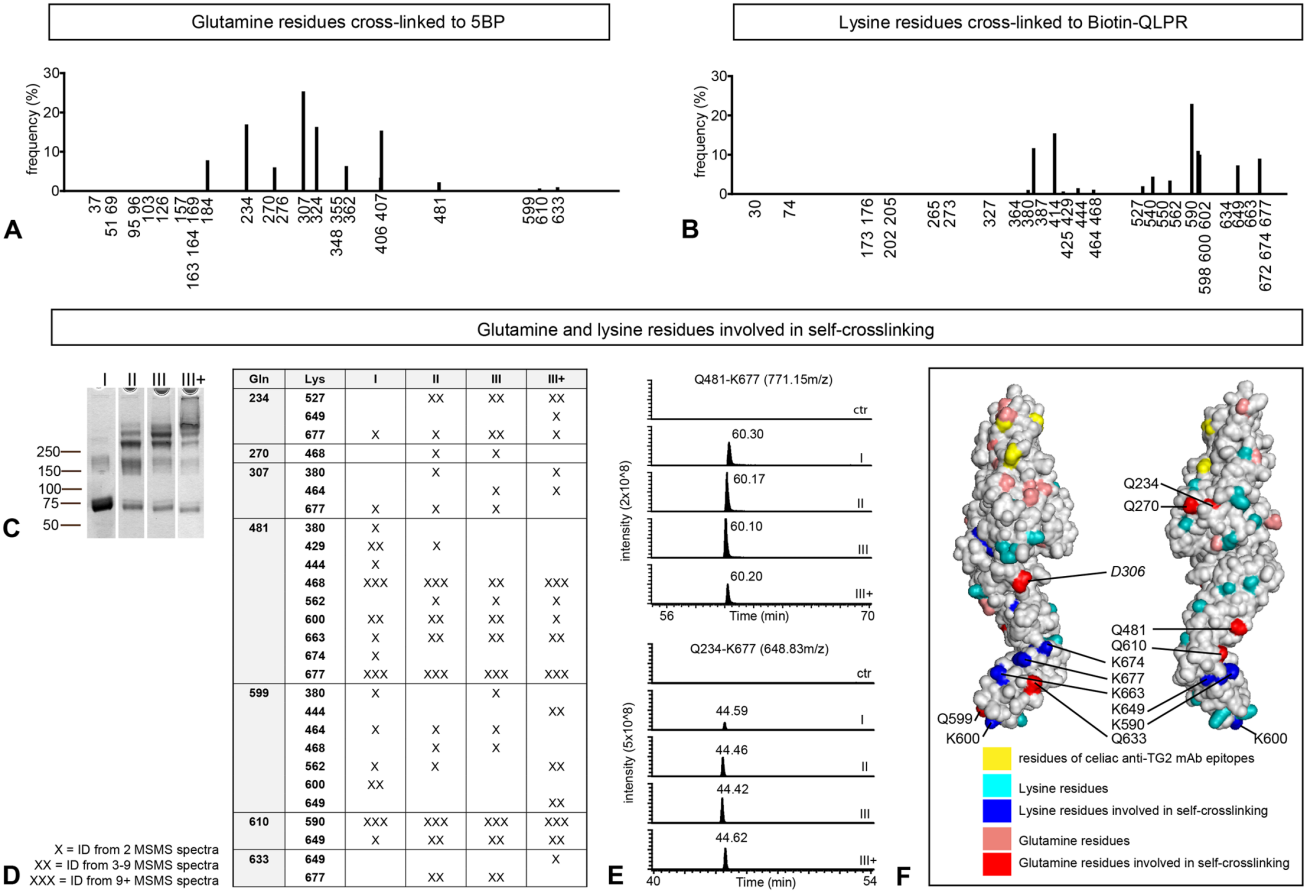
TG2-mediated self-crosslinking sites locate to the catalytic core and C-terminal part of TG2 (A) Glutamine residues of TG2 cross-linked to 5BP. The frequency (%) reflects the abundance of modified residues compared to each other. (B) Lysine residues of TG2 cross-linked to the small peptide Biotin-QLPR. Histograms in (A) and (B) are representative of two and three independent experiments, respectively. (D) Cross-linked peptide adducts identified from in solution tryptic digests of size separated fractions shown in (C). Multiple cross-linked peptide adducts were identified and only peptide adducts identified by two or more independent MSMS scans are listed in (D). Results from the second sample of triplicates are shown. (E) Extracted ion chromatogram for the peptide adducts Q481-K677 (AVKGFR-VGQSMNMGSDFDVFAHITNNTAEEYVC(acetamide)R, 771.15 m/z; EIC window 771.15–771.25 m/z,) and Q234-K677 (AVKGFR-VVSGMVNC(acetamide)NDDQGVLLGR; 648.83m/z, EIC window 648.83–648.93m/z). Signal intensity (y-axis) reflects peptide abundance. (F) Location of glutamine and lysine residues in open conformation TG2 (PDB ID code 2Q3Z). Glutamine residues identified in cross-linked peptide adducts are shown in red, the remaining glutamine residues are shown in pink. Lysine residues identified in cross-linked peptide adducts are shown in blue, the remaining lysine residues are shown in turquoise. Residues E8, R19, K30 and D94, important for recognition of celiac anti-TG2 monoclonal antibodies [26], are shown in yellow. Q307 is not resolved in the crystal structure, and D306 (italics) is highlighted instead. K464 and K468 are also located in unresolved parts.

To determine self-substrate sites in TG2, we incubated TG2 either with the small gluten peptide analog biotin-QLPR or 5BP followed by in solution trypsin digestion and mass spectrometry (MS) analysis. Several glutamine residues primarily in the catalytic core were cross-linked to 5BP (Fig 4A), of which two have previously been reported (Q234 and Q307;[23]). Using biotin-QLPR we identified lysine residues available for cross-linking (Fig 4B), and six of these have previously been identified [13]. From this it appears that multiple glutamine and lysine residues can act as self-substrate sites in TG2 when probed with small molecule substrates.

To identify glutamine and lysine residues involved in TG2-TG2 self-crosslinking, cross-linked TG2 was fractionated by size exclusion chromatography and fractions containing predominantly monomer/dimer (I), dimer/trimer (II), trimer/tetramer (III) or multimers (III+) (Fig 4C) were digested in solution with trypsin followed by nLC-MS/MS. Cross-linked peptides were identified using the software tool Stavrox [27]. Surprisingly, multiple cross-linked peptide adducts were repeatedly identified in each fraction. Those identified by two or more separate MSMS scans in a representative run are listed in Fig 4D. Involvement of multiple glutamine and lysine residue pairs suggests that there is a degree of redundancy to the self-crosslinking reaction. In keeping with this, mutation of four of the most predominant substrate glutamine residues (Q234N, Q307N, Q481N and Q610N) did not significantly affect self-crosslinking (S1 Fig). As expected, cross-linked peptide adducts harboring the mutated residues were no longer identified by MS (S1 Fig). The ability to utilize multiple residues for self-crosslinking corroborates the observation that gluten peptides are concomitantly incorporated into TG2 multimers without significantly affecting the multimerization reaction. This is also in line with the partial overlap between residues identified for TG2-TG2 cross-linking and residues modified with 5BP or Biotin-QLPR.

For several of the identified peptide adducts, we could compare abundance in the different fractions based on the extracted ion chromatograms for the identified m/z values (Fig 4E) although this was not possible for all peptide adducts. Some of the cross-linked peptides likely derive from intramolecular cross-links within the TG2 monomer (e.g. Q610-K590 and Q610-K649) in line with the fact that monomeric TG2 in CaCl_2_ treated samples migrates slightly faster than untreated TG2 in SDS-PAGE. Other cross-linked peptide adducts probably derive from intermolecular cross-links (e.g. Q234-K677 and Q481-K677). Presence of these in the monomeric fraction (I) is likely due to residual amount of TG2 oligomers (Fig 4C).

In summary, TG2 self-crosslinking appears to involve multiple glutamine and lysine residues predominantly situated in the catalytic core and C-terminal domains of TG2 (Fig 4F; cross-linked glutamine residues in red and cross-linked lysine residues in blue). Importantly, epitopes in the N-terminal part of TG2, important for recognition of celiac anti-TG2 monoclonal antibodies [26] (Fig 4F, yellow), should remain untouched in TG2 multimers.

### TG2 multimers efficiently activate TG2-specific B cells

**Fig 5:**
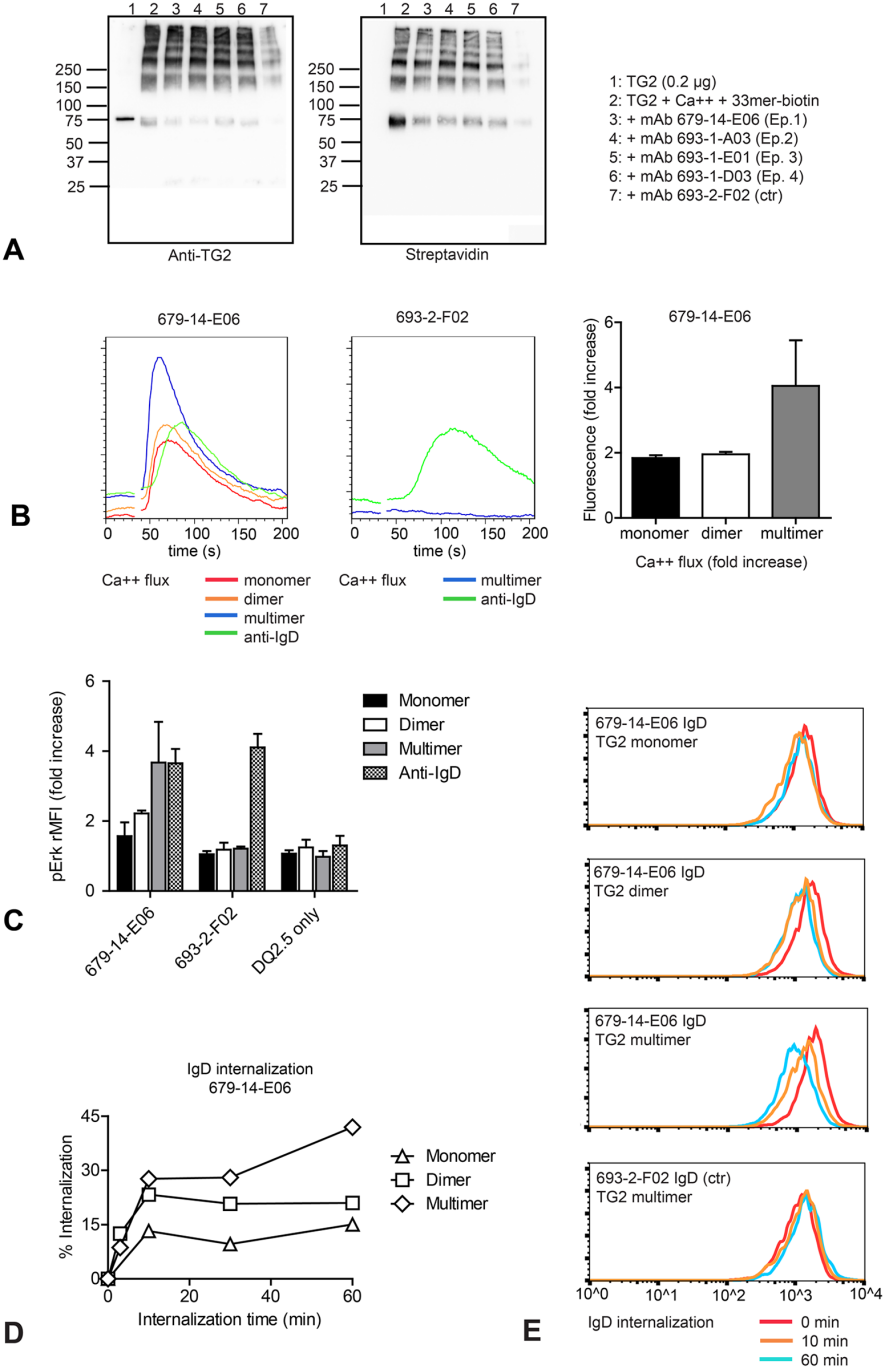
TG2 multimers are superior to TG2 monomer in activation of TG2-specific A20 B cells (A) Celiac TG2-specific monoconal antibodies are able to bind TG2-gluten multimer complexes. TG2 crosslinked in the presence of biotin-33mer peptide was immunoprecipitated using TG2-specific monoclonal antibodies representing all four celiac epitopes, or using a non TG2-specific monoclonal antibody (693-2-F02). TG2 was detected with mouse monoclonal antibody CUB7402 and TG2-bound peptide was detected with HRP-conjugated streptavidin. (B) Intracellular Ca^2+^ flux was compared following stimulation of transduced A20 B cells expressing HLA-DQ2.5 and TG2-specific (679-14-E06) or non TG2-specific (693-2-F02) IgD BCR with size-separated TG2 complexes or bivalent mouse anti-human IgD antibody. TG2 multimers were superior to monomer and dimers. Calcium flux induced by TG2 was BCR dependent and not observed in control cells. (C) A20 cells expressing TG2-specific (679-14-E06), non TG2-specific (693-2-F02) BCR or only HLA-DQ2.5 were incubated with complexes of TG2 and FITC-labeled 33mer peptide or with anti-human IgD antibody followed by staining for intracellular phoshorylated Erk (pErk). ERK phosphorylation was BCR-dependent and was not observed in A20 cells expressing only HLA-DQ2.5. (D, E) A20 cells expressing HLA-DQ2.5 and TG2-specific (679-14-E06) or non TG2-specific (693-2-F02) IgD BCR were incubated on ice with complexes of TG2 followed by incubation at 37°C. BCR internalization was assessed by flow cytometry staining for surface IgD. Columns in (B) show the means of triplicates from the second experiment (+SD), columns in (D) show the means of two independent experiments (+SD).

Conceivably, formation of TG2-multimers is relevant for activation of TG2-specific B cells in celiac disease, and incorporation of gluten peptides into multimers would allow efficient collaboration between TG2-specific B cells and gluten-specific T cells. A prerequisite for B-cell activation is recognition of the antigen by the BCR. To determine whether celiac anti-TG2 antibodies, and thus BCRs, could still recognize multimerized TG2 crosslinked to gluten peptide, we performed immunoprecipitation of TG2 incubated with CaCl_2_ in the presence of biotinylated 33mer gluten peptide using monoclonal human IgG antibodies derived from our panel of recombinant celiac anti-TG2 antibodies [14]. Antibodies of all four epitopes could bind and pull down TG2-TG2-33mer complexes (Fig 5A). Thus, the epitopes are conserved in TG2 multimers as predicted from our MS analysis.

We next addressed the effect of TG2 multimerization on B-cell activation and BCR internalization. To represent celiac TG2-specific B cells, we transduced murine A20 lymphoma cells with TG2-specific (679-14-E06) or non TG2-specific (693-2-F02) human IgD as BCR together with HLA-DQ2.5 [14]. As a measure of B-cell activation and BCR signaling we compared intracellular Ca^2+^ flux following incubation with antigens of different valency. TG2 multimers were indeed superior to fractions harboring predominantly TG2 monomer or TG2 dimer. TG2 multimers did not induce Ca^2+^ flux in non TG2-specific cells (693-2-F02) (Fig 5B). Similarily, flow cytometry staining for phosphoERK following incubation with TG2 monomer, dimer and multimers show that TG2 multimers were superior to monomer and dimer, and induce phosphoERK levels comparable to anti-IgD monoclonal antibody (Fig 5C). Non TG2-specific cells responded to BCR engagement by anti-IgD whereas incubation with TG2 had no effect. The increase in phosphoErk was BCR dependent as no increase was observed in A20 cells transduced only with HLA-DQ2.5. We then addressed internalization of IgD from the cell surface by flow cytometry. A20 cells were incubated with TG2 monomer, dimer or multimers, washed and moved to 37°C to permit internalization. Comparing remaining surface IgD staining with cells kept on ice to prevent internalization, we observed that TG2 multimers induced higher degree of internalization compared to monomer and dimer (Fig 5D and 5E). No IgD internalization was observed for non TG2-specific cells incubated with TG2 multimers. In summary, self-crosslinked multivalent TG2 is superior to monovalent TG2 both with regards to BCR signaling and BCR internalization, as addressed using transduced A20 B cells.

### In B-cell presentation of gluten-TG2 complexes to gluten-specific T cells, TG2-multimers are superior to TG2-monomers

**Fig 6:**
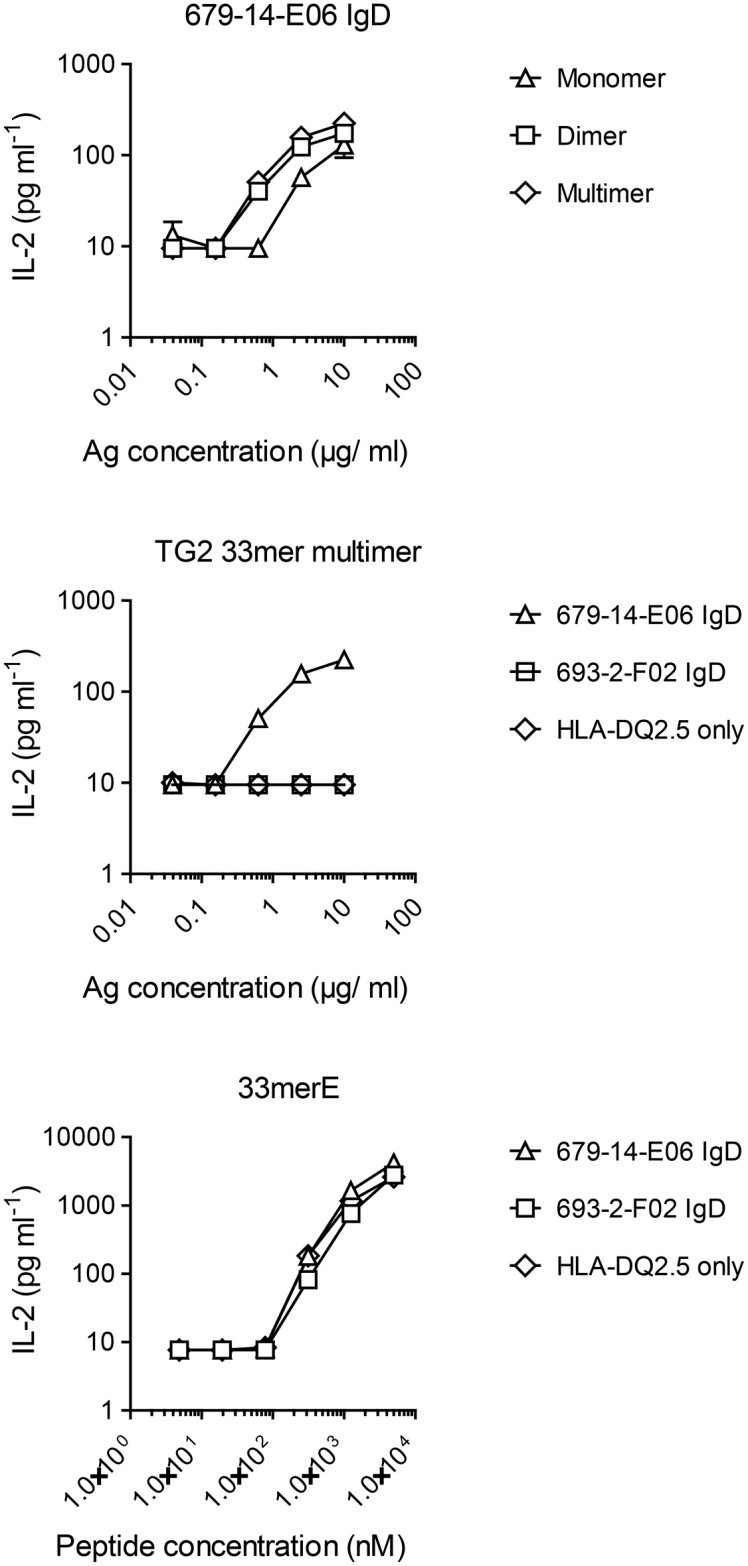
Presentation of gluten peptide by TG2-specific B cells upon incubation with TG2-gluten peptide complexes A20 B cells expressing HLA-DQ2.5 alone or in combination with TG2-specific (679-14-E06) or non TG2-specific (693-2-F02) IgD BCR were incubated with size-fractionated complexes of TG2 and FITC-33mer peptide, and their ability to present deamidated peptide to DQ2.5-glia-α2-specific hybridoma T cells was assessed by measuring release of IL-2. When incubated with the free, deamidated peptide (33merE), all A20 cells were capable of presenting peptide to the T cells. Only TG2-specific cells, however, could take up and present complexes of TG2 and peptide.

Help from gluten-reactive T cells following B-cell uptake of TG2-gluten complexes is likely crucial for the development of the anti-TG2 response in celiac disease. As valency of TG2 had a clear impact on B-cell activation, we next addressed if this also is reflected in increased activation of gluten-specific T cells. Size-fractionated TG2 cross-linked in the presence of FITC labeled 33mer gluten peptide, which harbors multiple copies of the DQ2.5-glia-α2 epitope, was offered to the transduced A20 lymphoma cells and T-cell activation was assessed by IL-2 production of a T-cell hybridoma expressing T-cell receptor specific for the HLA-DQ2.5-glia-α2 epitope. When offering equal amounts of antigen (in μg TG2), TG2-33mer dimer and multimer were more effective at inducing IL-2 production than TG2-33mer monomer (Fig 6). Importantly, T-cell stimulation was dependent on BCR internalization as no T-cell proliferation was observed using TG2 multimers with non TG2-specific cells. Self-crosslinked TG2 multimers harboring gluten T-cell epitopes are thus highly potent B-cell activators that also allow efficient uptake and presentation of gluten peptides to gluten-specific T cells.

## Discussion

We here report features of TG2 that potentially explain how this self-protein can be the target of the massive B-cell response observed in celiac disease. We have demonstrated that the enzyme readily cross-links itself and creates covalently linked self-multimers even at conditions with large excess of competing amine- or glutamine-containing substrates. Gluten peptides were readily incorporated into TG2-multimers. Indeed, TG2 multimers efficiently activated TG2-specific B cells compared to monomer, and TG2 multimers harboring gluten T-cell epitopes were efficiently internalized by TG2-specific B cells and presented to gluten-specific T cells. Thus, multimers of TG2 decorated with gluten peptides appear to represent an antigenic structure effective for breaking of B-cell tolerance in celiac disease, likely via extrafollicular or short duration germinal center reactions guided by high antigen avidity.

The immunoglobulin genes of TG2-specific plasma cells from the celiac lesion have relatively few somatic mutations [14]. The number of plasma cells and the level of anti-TG2 serum antibodies decrease rapidly upon removal of gluten from the diet [9, 15]. This could suggest that TG2-specific plasma cells derive from B cells that have been activated extrafollicularily. Upon antigen binding, B cells can either enter the germinal center for BCR affinity maturation through somatic hypermutation, or undergo rapid extrafollicular activation and plasma cell differentiation [18]. Using the hen egg lysozyme model system Paus and coworkers demonstrated that antigen avidity rather than BCR affinity for the epitope, is the determining factor in driving B cells towards an extrafollicular activation route [28]. Conceivably, activation of TG2-specific B cells by self-multimerized TG2 could promote extrafollicular B-cell activation or short duration germinal center reactions, explaining the observed features of TG2-specific plasma cells in the celiac lesion. An alternative model involves the selective activation of IgD-expressing, TG2-specific B cells outside of germinal centers through the ability of TG2 to cross-link IgD molecules to each other or to gluten peptides [14] (Iversen R et al, *manuscript submitted*). The two models, however, are not mutually exclusive and could both contribute to the activation of TG2-specific B cells at extrafollicular sites. Notably, though, the present model also may explain why the antibody response towards deamidated gluten in celiac disease shares the feature of low mutation rate [16] and short half-life of serum antibody titers, as the parallel incorporation of gluten peptides into TG2 multimers creates an antigen that could also activate the gluten-specific B cells. The fact that multiple glutamine and lysine residues are utilized as self-substrate sites explains why gluten peptides are readily incorporated also into the TG2-multimers. We observed that TG2-specific B cells efficiently presented gluten peptides to gluten-specific T cells when offered complexes of TG2 and gluten peptide, and preliminary experiments indicate that B cells with gluten-specific BCRs indeed efficiently bind multivalent TG2-gluten complexes and present gluten epitopes to gluten-specific T cells (du Pré MF, *unpublished observations*). Thus, the humoral response towards TG2 and gluten in celiac disease could be fueled by the same antigenic structure promoting generation of plasma cells with low mutation rate either via an extrafollicular route or via germinal center reactions of short duration.

It is well established that multivalent antigens are special with regards to antigen uptake and activation of B cells [19, 20, 29]. BCR multivalent engagement, first demonstrated by the difference between anti-immunoglobulin F(ab´)_2_ and Fab′ fragments [30], is dramatically more efficient at activating B cells than monovalent BCR engagement. In experiments where primary BCR transgenic B cells were treated with antigen, it was demonstrated that multimers (dimers, trimers and tetramers) of hen egg lysozyme formed by chemical cross-linking were equally efficient as monomers at eliciting early BCR signaling events, but much more efficient for antigen presentation to cognate CD4 T cells [20].

Multivalent antigens are particularly important for activating self-reactive B cells. In mice expressing soluble hen egg lysozyme in which B-cell tolerance is maintained by anergy [31], hen egg lysozyme-specific anergic B cells could be activated when the antigen was provided in a multimeric membrane bound form together with alloreactive T-cell help [32]. Similarly, hen egg lysozyme antigen when conjugated to multivalent virus-like particles induced autoantibody responses in transgenic mice expressing soluble hen egg lysozyme [21]. This was dependent on the presence of CD4 helper T cells specific for epitopes of the particle. Hence, antigen multivalency and T-cell help co-operate to reverse B-cell anergy. In view of this evidence, display of TG2 in multivalent complexes decorated with gluten peptides and involvement of gluten reactive CD4 T cells thus appears as an attractive mechanism to explain the breaking of B-cell tolerance to TG2 in celiac disease.

TG2 being a substrate for itself has been observed also for TG2 purified from tissues [33]. We find that the preference of TG2 for utilizing itself as a substrate relies on conformation-dependent TG2-TG2 association that involves the C-terminal domains of the protein. Binding of Ca^2+^ promotes this homotypic association with a progression from initial transient dimer/trimer formation to high molecular weight multimer formation at *in vitro* saturating concentrations of CaCl_2_. It is likely that transient dimer/trimer formation is sufficient to promote covalent self-crosslinking. The glutamine and lysine residues we identified as self-crosslinking sites are located in the catalytic core and C-terminal part of TG2, and gluten peptides were also predominantly cross-linked to lysine residues in the C-terminal half of TG2. In line with this, we observed that gluten-TG2 multimers are recognized by celiac disease patient-derived anti-TG2 monoclonal antibodies representative of the four major celiac disease TG2 epitopes located in the N-terminal half of TG2 [17]. This may be coincidental, but would also fit with a model wherein TG2 multimers drive the activation of TG2-specific B cells. In such multimers, only epitopes in the N-terminal part of TG2 would remain unchanged and available for BCR-binding.

At this point we are unable to test *in vivo* the implications of TG2 self-multimerization on breaking of B-cell tolerance and the generation of extrafollicular responses due to lack of appropriate models. Lupus disease causing extrafollicular B-cell responses were demonstrated in a knock-in transgenic mouse model for rheumatoid factor-specific BCR [34]. The multivalent nature of the antigen [35], as well as the ability of the immune complexes to give Toll-like receptor stimulation [36], was proven instrumental in this model. Attempts to establish a transgenic mouse model using BCR of a celiac disease anti-TG2 autoantibody is ongoing. Such an *in vivo* model would be instructive not least because we do not known where *in vivo* B cells encounter the TG2 antigen and, importantly, where *in vivo* TG2 is catalytically active.

In summary, the observation that the celiac autoantigen TG2 specifically catalyzes self-multimerization in a manner that preserves the dominant celiac anti-TG2 IgA epitopes is compelling as it may explain not only the low mutation rate and short lifetime of TG2 specific plasma cells, but also how, and why, the threshold for activation of self-reactive B cells can be reached in celiac disease. Future efforts will aim to determine the *in vivo* effects of such complexes and whether they can form and be detected in tissues.

## Materials and Methods

### Recombinant proteins

Recombinant human TG2 was either obtained from Phadia as purified protein produced in Sf9 insect cells or expressed in *E*. *coli* and purified as previously described [37]. Unless otherwise indicated, Sf9 produced TG2 was used for all experiments. Single site mutants and TG2 deletion mutant was produced in *E*. *coli*. Mutations were introduced using the QuickChange Multi Site-Directed Mutagenesis Kit (Stratagene). Purified inactive inhibitor-bound TG2 or TG2 1–465 was obtained by incubation with active site inhibitor DP3-3 (Ac-P-DON-LPF-NH_2_) at a 10:1 weight ratio (Zedira) in the presence of CaCl_2_ followed by strong cation exchange purification. TG2-specific human monoclonal IgG antibodies were produced as previously described [14]. Briefly, IgG1and Igκ expression vectors containing the cloned heavy and light chain variable regions were co-transfected into HEK293 cells and supernatants day 6 post transfection were purified on Protein G Sepharose (GE Healthcare).

### TG2 self-cross-linking and SDS-PAGE

TG2 crosslinking reactions were performed at 37°C in TBS pH 7.4 (50 mM Tris HCl, 150 mM NaCl). For separation of cross-linking products, TG2 was incubated with 5 mM CaCl_2_, 1 mM EDTA or 1 mM active site inhibitor KCC009 together with 5 mM CaCl_2_. Effect of exogenous substrate was addressed by incubation of TG2 (1 μM) with 1 mM or 5 mM CaCl_2_ in the presence or absence of FITC-labeled gluten peptides DQ2.5-glia-α2(QQ) or DQ2.5-glia-α2(QE) (FITC-Ahx-PQPQLPYPQPQLPY and FITC-Ahx-PQPQLPYPQPELPY respectively; GL Biochem) and 5BP (ThermoFischer). Effect of exogenous protein was addressed by incubation of TG2 (2 μM) with 5 mM CaCl_2_ in the presence of human albumin, chicken ovalbumin and human IgG (all from Sigma, 0.2 μg/μl each) for 30 min and incubation of TG2 (1.3 μM) in the presence or absence of the 29kDa FN proteolytic fragment (9.3 μM, Sigma Aldrich) for 10 and 20 min. Crosslinking of *E*. *coli*-produced full length TG2 and TG2 1–465 deletion mutant was compared following 60 min incubation using 0.7 μM, 1.3 μM and 4.2 μM enzyme and 1.3 μM enzyme in the presence of 0.2 mM FITC-DQ2.5-glia-α2(QQ). Reactions were stopped either by addition of 5 mM iodoacetamide or 4x Laemmli sample buffer followed by heating in the presence of β-mercaptoethanol. Proteins were separated on 4–20% TGX gels (BioRad) unless otherwise indicated. Gels were stained with Biosafe Coomassie Stain (BioRad) and cross-linked FITC-labeled peptides were detected using a Kodak Image Station 4000MM Pro instrument. For mass spectrometry identification of 5BP or Biotin-QLPR (GL Biochem) modified residues, TG2 was incubated with 0.5 mM 5BP at 37°C for 25 min or with 0.2 mM Biotin-QLPR for 12 min. The reaction was stopped with 5 mM iodoacetamide and excess 5BP or Biotin-QLPR was removed by spin column separation prior to trypsin digestion.

### Dynamic light scattering analysis

*E*. *coli* produced inhibitor-bound TG2 (TG2-DP3-3 or 1-465-DP-3-3) was incubated at 0.2 mg/ml in 20 mM Tris, pH 7.2, 150 mM NaCl with 1 mM EDTA or indicated concentrations of CaCl_2_ at 37°C. Recordings were collected following a 2 min pre-equilibration step at different time-points using a Malven Zetasier Nano laser light scattering system (Malven). The measured volume percentage is reported.

### BS3 capture of non-covalent multimers

Due to the reactivity of Tris with the BS3 reagent, capture of non-covalent multimers was done using TG2 preparations that had been buffer-exchanged into 20 mM HEPES, pH 7.5, 150 mM NaCl using Zeba Spin columns (Pierce). TG2 with or without the active-site inhibitor DP3-3 was incubated in the presence or absence of 5 mM CaCl_2_ or 1 mM GTP for 30 min at 37°C or at room temperature followed by the addition of BS3 (Pierce) to a final concentration of 0.5 mM and additional incubation for 15 min at 37°C or 30 min at room temperature. The reaction was stopped by addition of 1 M Tris-HCl, pH 8.0, and the degree of cross-linking was assessed by SDS-PAGE analysis. In order to study the effect of the C-terminal domains of TG2, *E*. *coli* produced full-length TG2 and TG2 1–465 were compared in BS3-mediated cross-linking of the DP3-3-treated proteins at 37°C.

### Size exclusion chromatography

Self-crosslinked TG2 generated in the absence or presence of FITC-labeled 33mer (FITC-Ahx- LQLQPFPQPQLPYPQPQLPYPQPQLPYPQPQPF, GL Biochem) was fractionated on a Superdex 200 10/300 GL column (GE Healtcare) using TBS pH 7.4 with 1 mM EDTA as running buffer. Size-separated fractions were pooled to harbor predominantly monomer TG2 (monomer), dimeric/ trimeric TG2 (dimer) or multimeric TG2 (multimer), up-concentrated and quantified by Nano-drop using the Extinction Coefficient and molecular weight of monomeric TG2. The amount of cross-linked FITC peptide was quantified by comparing UV absorbance at 495 nm to FITC-labelled 33mer peptide of known concentration. All fractions had comparable amount of peptide incorporated per TG2 monomer.

### MS analysis and peptide identification

TG2 modified with 5BP or biotin-QLPR (GL Biochem) or self-crosslinked TG2 fractionated by size exclusion chromatography was reduced with dithiothretiol and alkylated with iodoacetamide followed by overnight digestion with sequencing grade trypsin (ProMega). Tryptic digests were purified on homemade C18 microcolumns prior to separation by nano-LC connected to a quadrupole-Orbitrap (QExactive) mass spectrometer (ThermoElectron). Samples were separated on 25 cm C_18_ columns using a linear gradient from 5–50% methanol and 0.1% formic acid from 0–120 min. Mass spectra were acquired using Xcalibur 2.2 software performing single MS full-scan (300–1750 m/z, 70,000 resolution at m/z 200) followed by 10 data-dependent MS/MS scans. For identification of tryptic peptides modified with 5BP or biotin-QLPR, database search was performed by MaxQuant (1.5.1.2) using a fasta-file of the TG2 sequence as in-house database and 5BP or biotin-QLPR as variable glutamine and lysine modifications respectively. To compare the abundance of 5BP-modified glutamine residues or biotin-QLPR-modified lysine residues, the intensities of identified peptides for each modified residue were summed and divided by the total intensity of all modified residues. For identification of cross-linked peptide adducts, in-house Mascot database search was performed using Mascot Distiller Daemon and a fasta-file of the TG2 sequence as database, allowing for 6 miscleavages of trypsin. Unmatched spectra were exported as mascot generic files (.mgf). These files were uploaded for database search using Stavrox (3.4.11) to identify cross-linked peptide adducts where glutamine to lysine isopeptide cross-linking was added as cross-linker. From each fraction, peptide adducts that obtained a score above an FDR of 0.01 were grouped and adducts that were identified more than once (i.e. with more than one MSMS spectrum) are presented in Fig 4D. This table displays results from a representative analysis of one sample set and is representative of several independent experiments (reanalysis of the same samples, analysis of different digests of the same protein fractions and digestion of different cross-linked fractions) that gave comparable results with regards to targeted glutamine and lysine residues albeit with some variation in scoring and frequency of identified peptide adducts. Notably, peptides harboring cross-links within the same peptide, or peptides harboring more than one cross-link would not be identified by this method. Extracted ion chromatograms were generated in the Xcalibur Qual Browser.

### Immunoprecipitation of TG2 multimers by celiac disease anti-TG2 antibodies

TG2 (0.3 mg/ml) was incubated with 0.1 mM biotin-labeled 33mer in TBS with 5 mM CaCl_2_ for 40 min at 37°C. The reaction was stopped by addition of EDTA, and monoclonal anti-TG2 antibodies (1 μg; 679-14-E06, 693-1-A03, 693-1-E01, 693-1-D03 or 693-2-F02; non TG2-specific) were added to the preformed TG2 multimers (0.4 μg). Samples were incubated for 30 min at 37°C and captured with Protein G dynabeads (Life Technologies). Bound proteins were eluted with 4xLaemmli sample buffer followed by SDS-PAGE (4–20% TGX) and semidry transfer to nitrocellulose membrane. TG2 was detected using anti-TG2 mouse monoclonal antibody CUB7402 (ThermoFishcer) followed by goat-anti-mouse-HRP (Southern Biotech) and enhanced chemiluminescence detection. The membrane was stripped and probed for IgG using rabbit-anti-IgG and rabbit-anti-kappa followed by goat-anti-rabbit-HRP (data not shown). The membrane was stripped and biotin-labeled 33mer was detected using streptavidin-HRP (Southern Biotech).

### Generation of transduced cell lines

A20 lymphoma cells expressing TG2-specific (679-14-E06) BCR or non TG2-specific (693-2-F02) BCR, together with human MCH class II HLA-DQ2.5 were generated and maintained as previously described [14]. The T-cell line BW58α-β- which lacks endogenous T-cell receptor was engineered to express human CD4 and the T-cell receptor derived from a DQ2.5-glia-α2-reactive CD4 T-cell clone (TCC364.1.0.14) as previously described [38].

### Intracellular Ca^2+^ flux

The intracellular free Ca^2+^ concentration in transduced A20 cells was determined using the Fluo-4 Direct Calcium Assay Kit (Life Technologies) according to the manufacturer’s recommendations. A20 cells were equilibrated to 37°C in RPMI supplemented with 10% FCS before an equal volume of 2x Fluo-4 Calcium Assay Reagent (Life Technologies) was added. Cells were incubated for 1.5 h at 37°C, washed 2x in RPMI/10% FCS and resuspended in 250 μl RPMI/10% FCS supplemented with 0.2 mM Probenecid (Life Technologies). Baseline Ca^2+^ was established by measuring 30 seconds on a FACSCalibur flow cytometer (BD Biosciences) before cells were stimulated with 2 μg/ml of TG2 monomers, dimers or multimers or biotinylated anti-human IgD antibody (IADB6, Southern Biotech). Calcium curves were read for 3 min. Kinetic curves were generated using FlowJO software (TreeStar).

### Flow cytometry staining of phosphoERK

A20 cells were incubated at 1 million cells per ml in the absence of antigen or together with 2.5 μg/ml biotinylated mouse anti-human IgD (IADB6, Southern Biotech) or 2.5 μg/ml of size-separated TG2- FITC-33mer gluten complexes (monomer, dimer or multimer) in 10% FCS/RPMI for 2 min at 37°C. The cells were immediately fixed by addition of formaldehyde to 4% (vol/vol) and incubated for 20 min at room temperature before they were lyzed in ice cold methanol, followed by incubation for 1h at -80°C. The cells were then stained with rabbit anti-phosphorylated Erk (D13.14.4E, Cell Signaling) followed by Alexa647-conjugated goat anti-rabbit IgG (Life Technologies) in 2% FCS/PBS and analyzed by flow cytometry using a FACSCalibur instrument (BD Biosciences). Signals from treated cells were compared to signals from untreated cell samples.

### BCR endocytosis

A20 cells were pulsed with 1 μg/ml of TG2 monomers, dimers or multimers on ice for 30 min and then washed. The pulsed cells were incubated at 37°C for the indicated time, returned to ice and fixed with 2% formaldehyde. No internalization control cells were left on ice during the whole period. The cells were stained with APC-labeled anti-human IgD (clone IA6-2; BD Biosciences) for 20 min on ice, washed and analyzed by flow cytometry using a FACSCalibur instrument. The mean fluorescence intensity (MFI) was determined and the percentage internalization was calculated by (MFI no internalization—MFI sample)/MFI no internalization x 100%.

### T-cell stimulation assay

Release of IL-2 by gluten-reactive hybridoma T cells after incubation with A20 B cells was used as a measure of T-B cell collaboration. To compare the ability of different transduced A20 cells to present peptide to the T cells, 5x10^4^ A20 cells expressing HLA-DQ2.5 alone or in combination with TG2-specific (679-14-E06) or non TG2-specific (693-2-F02) IgD BCR were incubated with various concentrations of deamidated 33mer peptide for 3 h at 37°C in 5% FCS/RPMI, followed by addition of 2.5x10^4^ hybridoma cells and incubation for additional 18 h. Secreted IL-2 was measured in the supernatant by ELISA as previously described [38]. To assess the ability of the A20 cells to present peptide after uptake of TG2-gluten complexes, the cells were incubated with various concentrations of complexes separated into monomer, dimer and multimer fractions for 20 min at room temperature in RPMI using 10 million cells per ml. The cells were then washed with RPMI and resuspended in 5% FCS/RPMI followed by incubation for 3 h at 37°C. 2.5x10^4^ hybridoma cells were added to 2x10^5^ antigen-pulsed A20 cells, followed by incubation and measurement of IL-2 as described above.

## Supporting Information

S1 Fig Redundancy of TG2 self-crosslinking reaction as observed by glutamine residue mutation (A) Mutation of the glutamine residues Q234, Q307, Q481 and Q610 did not significantly affect TG2 self-crosslinking as compared to wild type (WT) TG2 (1; no CaCl_2_, 2; 0.25 mg/ml enzyme with 5 mM CaCl2 37°C for 30 min, 3; as 2 with addition of 0.2 mM DQ2.5-glia-α2(EQ)). (B) Analysis of tryptic digest of cross-linked *E*. *coli* produced WT TG2 and a mutant (Mut) with Q234, Q307, Q481 and Q610 converted to asparagine. Cross-linked enzyme was not fractionated by size prior to trypsin digestion and analysis. The top 14 unique peptides are shown for WT and top 10 for Mut as fewer cross-linked peptides were identified in this sample. X denotes identification by one MSMS scan, XX denotes identification by 2 MSMS scans.
(TIF)
