# Supplementary figures and images for: Enhanced B-Cell Receptor Recognition of the Autoantigen Transglutaminase 2 by Efficient Catalytic Self-Multimerization
Source: PLoS One. 2015 Aug 5;10(8):e0134922. doi: 10.1371/journal.pone.0134922 (PMC4526674; doi:10.1371/journal.pone.0134922)

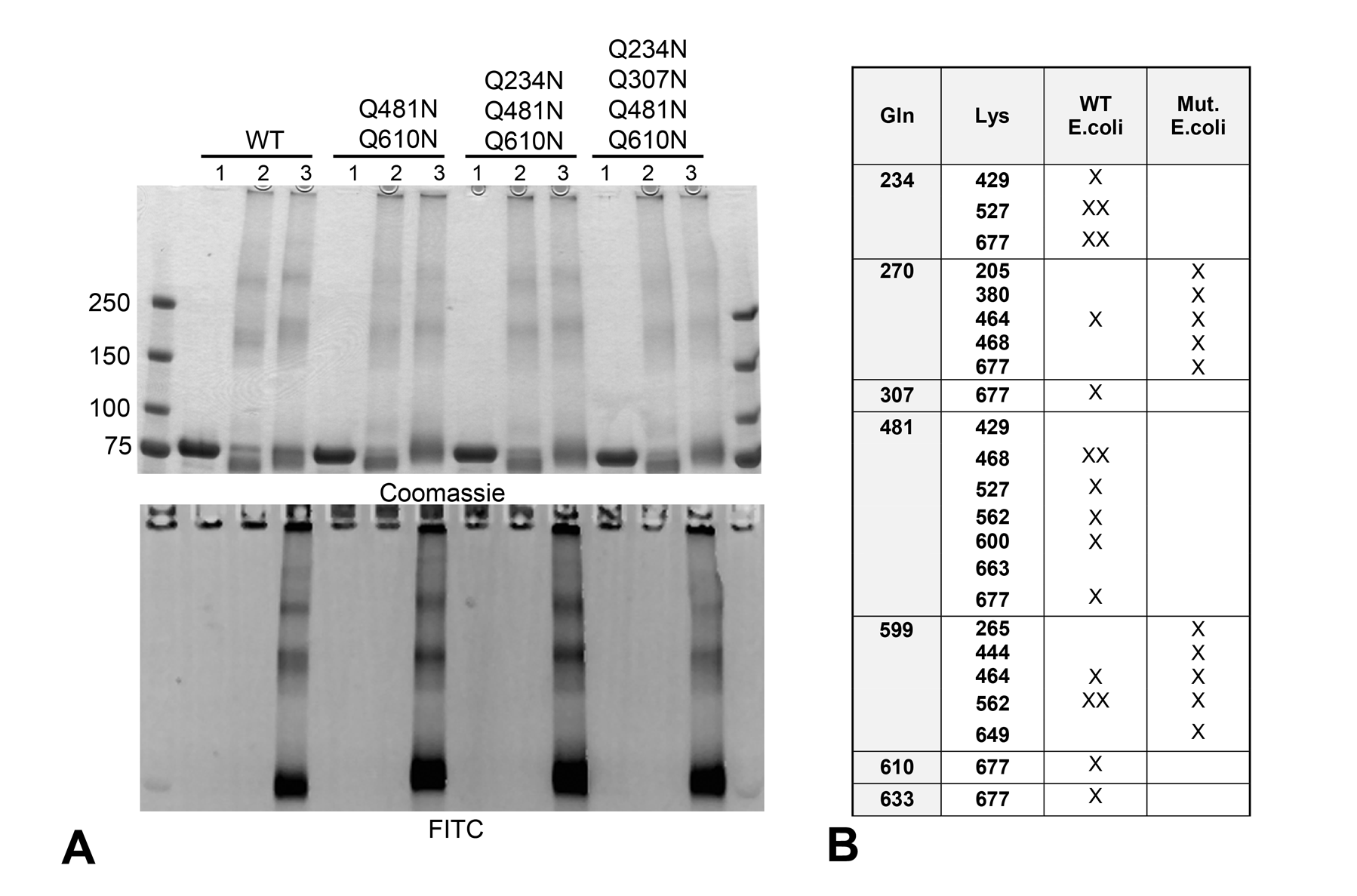

Supplement: S1 Fig — (A) Mutation of the glutamine residues Q234, Q307, Q481 and Q610 did not significantly affect TG2 self-crosslinking as compared to wild type (WT) TG2 (1; no CaCl2, 2; 0.25 mg/ml enzyme with 5 mM CaCl2 37°C for 30 min, 3; as 2 with addition of 0.2 mM DQ2.5-glia-α2(EQ)). (B) Analysis of tryptic digest of cross-linked E. coli produced WT TG2 and a mutant (Mut) with Q234, Q307, Q481 and Q610 converted to asparagine. Cross-linked enzyme was not fractionated by size prior to trypsin digestion and analysis. The top 14 unique peptides are shown for WT and top 10 for Mut as fewer cross-linked peptides were identified in this sample. X denotes identification by one MSMS scan, XX denotes identification by 2 MSMS scans. (TIF) [file pone.0134922.s001.tif]
